# Perspectives on Microbial Electron Transfer Networks for Environmental Biotechnology

**DOI:** 10.3389/fmicb.2022.845796

**Published:** 2022-04-12

**Authors:** Shaofeng Zhou, Da Song, Ji-Dong Gu, Yonggang Yang, Meiying Xu

**Affiliations:** ^1^Guangdong Provincial Key Laboratory of Microbial Culture Collection and Application, State Key Laboratory of Applied Microbiology Southern China, Institute of Microbiology, Guangdong Academy of Sciences, Guangzhou, China; ^2^Environmental Science and Engineering Group, Guangdong Technion-Israel Institute of Technology, Shantou, China

**Keywords:** electroactive microorganisms, electromicrobiology, biological treatment, synthetic microbiome, microbial electron transfer networks

## Abstract

The overlap of microbiology and electrochemistry provides plenty of opportunities for a deeper understanding of the redox biogeochemical cycle of natural-abundant elements (like iron, nitrogen, and sulfur) on Earth. The electroactive microorganisms (EAMs) mediate electron flows outward the cytomembrane *via* diverse pathways like multiheme cytochromes, bridging an electronic connection between abiotic and biotic reactions. On an environmental level, decades of research on EAMs and the derived subject termed “*electromicrobiology*” provide a rich collection of multidisciplinary knowledge and establish various bioelectrochemical designs for the development of environmental biotechnology. Recent advances suggest that EAMs actually make greater differences on a larger scale, and the metabolism of microbial community and ecological interactions between microbes play a great role in bioremediation processes. In this perspective, we propose the concept of microbial electron transfer network (METN) that demonstrates the “species-to-species” interactions further and discuss several key questions ranging from cellular modification to microbiome construction. Future research directions including metabolic flux regulation and microbes–materials interactions are also highlighted to advance understanding of METN for the development of next-generation environmental biotechnology.

## Introduction: Microbial Electron Transfer Network

Near one hundred and a half years ago when Thomas Edison devoted himself to the improvement of light bulbs, he would probably never imagine that some of the bulbs could be powered by a bunch of tiny microbes named electroactive microorganisms (EAMs). After a rapid development in the past decade, scientists have contributed worthwhile endeavors to establish the scientific basis of potential microbial electrochemical technologies such as microbial fuel cells or microbial electrolytic cells to deal with various environmental issues (Zhang and Angelidaki, 2014; Gude, 2016). Up to this day, the scope of “*electromicrobiology*” is far beyond microbial electron exchange with electrodes, but extended significantly to the microbe-mediated redox reactions between microbes, as well as between microbial cells and the ecosphere, driving both biotic and abiotic natural element cycles (Nichols et al., 2015; Marzocchi et al., 2022). For instance, methanogens as ancient microbes were suggested to be responsible for annually producing one billion metric tons of methane in the global carbon cycle, taking micromolecular carbon chemicals (i.e., acetate) as terminal electron acceptors (Prakash et al., 2019). Furthermore, some methanogens were recently evidenced to transfer electrons to exogenous Fe(III) for conserving energy, which was important in the early evolution of respiration (Lueders and Friedrich, 2002; Seckbach, 2004; Prakash et al., 2019). Beyond that, there are other EAMs unintentionally influencing and shaping our blue planet Earth in totally different ways like mineral diffusion in soil or sediments (Lovley et al., 2011). In particular, the recent findings on cable bacteria and filamentous bacteria have revolutionarily expanded the wide spectrum of bio-electron flow from micrometer to centimeter scale, cooperatively connecting assorted biochemical reactions [e.g., sulfate oxidation, reduction of dissolved oxygen, and Fe(II)/Fe(III) transformation] from anoxic to oxic conditions in sediments (Liu et al., 2021a; Yang et al., 2021). On this topic, a microbial electron transfer network (METN), which is a three-dimensional collection of extracellular electron transfer (EET) behaviors among aggregative microflora of the phylogenetically diverse EAMs and even non-electroactive ones, is certainly evident and ubiquitous in both natural and engineering environments. In the viewpoint of environmental bioremediation, it continues to make us wonder if it is possible that the development of biotreatments could be guided by the theories of METN, which had been less focused on previously. We hope that this idea of METN could help the scientific community bring a deeper understanding to answer the following two fundamental questions: (1) How does one modify and optimize functional microbiome including EAMs and non-electroactive ones in a real environment? (2) To what extent could this artificial modification be regulated for the environmental biotechnology development?

## Discussion

### Why Is Microbial Electron Transfer Network Important?

On a cross-sectoral scale, environmentalists may be more interested in how the METN works in some biological treatment processes, e.g., membrane bioreactor and anaerobic digestion, especially for granular sludge-based structures in which microbiome shares micro-niche intimately and intensive mass-transfer flow both individually and collectively. Taking anaerobic digestion as an example, besides either H_2_ or acetate as electron donors for methanogenesis processes, direct interspecies electron transfer (DIET) is recognized as a highly efficient and stable process connecting both respiratory and fermentative bacteria/archaea for bioconversion from (macro-molecular) organics to methane (Lovley and Holmes, 2021). Speaking of which, the fast development and broad application scenarios of genomics could provide plenty of correlative information from other transboundary research. For instance, a two-species microbial coculture was established for value-added chemical evolution driven by bio- and light energy (Huang et al., 2022). In this case, *Rhodopseudomonas palustris* harvested and transferred solar energy into bioenergy (bio-electrons) while the other, *Methanosarcina barkeri*, conducted CO_2_-to-CH_4_ conversion powered by the bio-electron flow. The key question was how those bio-electrons passed through cytomembranes. Revealed by metatranscriptomic analyses, both multihaem cytochrome c and nanofilaments (direct contact) and electron shuttles (indirect connect) wired two species to construct a biological hybrid system (Huang et al., 2022). Coincidentally, a model of DIET between acetate-consuming bacteria and methanogens was recently established *via* genome-centric metatranscriptomics analysis; either electrically conductive pili (e-pili) and cytochromes or artificial materials (hydrochar) were evident as available electric conduits for DIET (Shi et al., 2021). Those intriguing findings, of course, would be helpful and constructive for environmentalists to optimize related environmental biotechnology.

Now, back to the question of whether METN is important for future environmental biotechnology. More than 400 scientific papers per year have been published within the broad scope of ‘‘*biological treatment*’’ and ‘‘*electron transfer*’’ in the past 5 years (based on the web search results^1^). The answer is obvious. New scientific discoveries and insights have expanded upon new electroactive species and novel electric bridges; however, a coherent and comprehensive picture or framework of METN on how it works in biological applications and its associated environmental implications is still not available. It would be of great significance to formulate this concept to guide the construction and operation of biological treatments from lab-scale to real application, in a multidiscipline view.

### In What Area Could Microbial Electron Transfer Networks Be Improved?

As a manifestation of functional microflora behaviors, cell performance in METN is paid more attention prior to METN improvement in the eyes of environmentalists. For example, it is known that the electric conduits of EAM are arrayed disjunctively on the cell surface (Lovley and Holmes, 2021). This fact, however, makes the bioelectric connection of e-pili or other EET-related proteins with extracellular electron barriers/terminals become random behaviors that require close contact and more active interfacial area. In this sense, METN is fundamentally an issue of mathematic probability. The fast-growing literature has suggested “top-down” strategies. Theoretically, the inactive electric conduits on a single-EAM cell could be wired up by covering abiotic conductive electron collectors (e.g., FeS or polydopamine), achieving record-high EET efficiency (Yu et al., 2020). In this way, it is an encouraging story in which EET had been evolved from natural “dot-to-dot” contact to artificial “cell sphere-to-cell sphere” connection. Recently, this story is enriched by another article published in *Science*. The *Shewanella* sp., a famous EAM model strain, was *in vivo* embedded with silver nanoparticles for excellent fuel-utilization efficiency in microbial fuel cells (Cao et al., 2021). Although they are followed by universal controversies, the presented findings strongly imply that the cytomembrane-level deficits are responsible for the sluggish electron transfer efficiency. However, the cytomembrane-level modification is still not “three-dimensional” enough before its competing mechanisms on “species-to-species” connections in METN uncovered from mysteries. To this end, some redox-active substances (e.g., elemental sulfur) are suggested to be useful for mediating fundamental interactions on the species level (Zhang et al., 2021). Those redox-active substances (or generally called electron shuttles) could act as driving forces for indirect interspecies electron transfer (IIET). IIET was also intensively researched in fields like iron cycle in sedimentary environments, anaerobic digestions, and microbial electrosynthesis (Weber et al., 2006; Rabaey and Rozendal, 2010; Shi et al., 2016). It could be a critical framework of METN that makes the “microcosm-to-microcosm” communication possible. However, unlike DIET, progress on IIET has been slow to sufficient, considering that the vast candidates for electron shuttles ranged from artificial additives (e.g., H_2_, biochar, and flavins) to microbial secreta (e.g., soluble *c*-type cytochromes) (Liu et al., 2020; Wu et al., 2020; Zavarzina et al., 2020). Overall, such “top-down” strategies mainly focus on modifying and optimizing the natural-given properties of EAM, whereas how to piece together those nature-given properties is still a fundamental question awaiting an answer.

**FIGURE 1:**
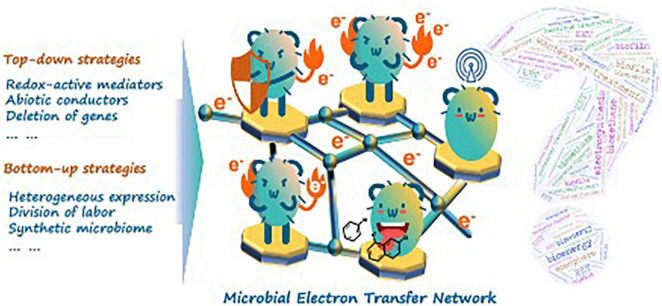
METN development for next-generation environmental biotechnology.

Though great strides have been made, an ideal niche for METN remains difficult to maintain in real biotreatments. It was found that the interspecific competition even within the same genus (i.e., *Geobacter* spp.) would largely alter the electron transfer networks in complicated microbial consortia (Yan et al., 2021). Here, we also propose bottom-up strategies on research of the eco-niches and microbial interaction of METN in artificial and engineering biosystems for fundamental and practical interests. For example, the synthetic microbiome, a rationally programmed microbial consortia with engineering strategies (i.e., quantification, standardization, and modularization) into the assembly of functional microbiome, opens a modular toolbox for scientists to break the limitation of natural evolution of METN (Lawson et al., 2019; Jaiswal and Shukla, 2020). In other words, we can now create a synthetic METN microcosm with known microbial consortia (Figure 1). Recently, a synthetic METN microcosm within a three-species microbial consortium (engineered *Escherichia coli*, *Bacillus subtilis*, and *Shewanella oneidensis*) was constructed following a “division-of-labor” principle, resulting in better bioenergy generation during which the production of electron donors/shuttles and bioenergy recovery were separately allocated in the three-species microbial consortium (Liu et al., 2017). The principle was generally exerted and tested through a cross-feeding strategy in most research. For example, it was found that chromate [Cr(VI)] could be reduced in an anaerobic digestion sludge coupled with elemental sulfur [S(0)] or zerovalent iron [Fe(0)] as the electron donor (Shi et al., 2019). This process was mediated by a typical cross-feeding strategy. The volatile fatty acids produced by S(0)- or Fe(0)-oxidizing bacteria (like *Thiobacillus* spp. and *Ferrovibrio* spp., respectively) could be used to further metabolize the chromate-reducing bacteria (like *Geobacter* spp. or *Desulfovibrio* spp.). This finding was quite important as it provided a potential microbial consortia design for Cr(VI) removal in groundwater and other water streams where proper organic electron donors are insufficient and any treatments that potentially bring secondary pollution are strictly forbidden. Though promising, the scientific community may expect a database-like toolbox recording interspecific synergy or even competition to better advise the research and application attempts of the synthetic METN microcosm. Ignoring immense technology transfer issues, it is not surprising to expect that the METN effectiveness could be largely improved and dynamically controlled by means of constructing synthetic microbial consortia with designed objectives.

### Future Research Directions

Though successful examples have been introduced above, the metabolic pathway segregation for establishing rational “division of labor” should be noted for further verification and implementations. Taking METN as a whole bio-unit in biological treatments, how to regulate metabolic flux (mass, energy, and information flows) in METN to avoid unwanted selection bias is a huge challenge. To answer this, cytocompatible EET circuits and eco-compatible strains should be carefully constructed, selected, and assembled in the METN microcosm. Though most proposals of “cytocompatible EET” establishment are based on the modifications of electroactive species, the unique EET capability of EAMs may place themselves as potential chassis cells for “eco-compatible strains” construction to deeply modify the metabolic flux (especially energy flow) in such biotechnology (synthetic biology). In this case, biosafety should be carefully noted in the research to avoid the diffusion of modified genes into the natural gene pool. Nevertheless, intensive “Design–Build–Test–Learn cycles” should be both essentially and iteratively conducted for objective-driven and precise optimization. Strategies including competition-related elements (e.g., toxin secretion systems) and cross-feeding can be applied to maintain the balance of this synthetic METN microcosm. Though it seems impossible to universally know the specific functions of each microorganism, piecing together clues behind the mechanisms of METN would be a potential shortcut. Speaking of which, quorum sensing (QS) could be probably taken into consideration on the microbial compatibility control. QS is basically an interspecies communication process during microbial aggregation, biofilm formation, and granulation, induced by a series of QS signals like homoserine lactones and autoinducing peptides (Maddela et al., 2019). Of most interest, the signals could increase the concentration and redox activities of extracellular polymeric substances from electroactive biofilm (Chen et al., 2017). Here, we appeal for more efforts on microbial interactions (synergy, mutualism, competition, etc.) of METN, especially on connections between key functional METN microbiomes and non-electroactive species.

Microbial electron transfer network is clearly not an exclusive concept of EAMs or other microorganisms; more innovative research on the microbes–materials interactions should be conducted both technically and economically to increase the knowledge base and the competitiveness of related environmental biotechnologies. In particular, questions on how to expand the influence range of METN and to what extent could METN be domesticated still perplex environmental researchers. Energy taxis, as a key branch of chemotaxis, was recently proposed to control the transport and motility of *S. oneidensis* MR-1 in porous media (Liu et al., 2021b). It is important as the migration of those functional microbes could hopefully be controlled toward (micro)pollutant sources along gradient redox-active material surfaces. Such effectiveness provides a complementary balance strategy between two major contradictions: excessive population growth and biomass running off. Either overpopulation or flushing loss of biomass is disfavored in environmental biotechnology since it would cause severe sludge accumulation and inexorable crash in efficiency, resulting in added complications on reactor operation. Thus, harnessing energy taxis to different redox materials could be effective for METN regulation, but relevant research is still in its infancy. Biochar is also an excellent candidate for METN regulation as its sources are earth-abundant, and importantly, it is highly redox-active with sufficient micro- and macropores (Mohanty and Boehm, 2014; Zhou et al., 2019). Attempts have been made to substantially expand the electronic reach of METN by wheat straw-derived biochar for bioremediation of pentachlorophenol-contaminated soils (Cai et al., 2020). On the other hand, once it involves usage of materials, the operation cost should be calculated and reported (Zhang and Angelidaki, 2016). We are expecting more voices and more research activities on this topic for further advances made in the near future.

## Data Availability Statement

The raw data supporting the conclusions of this article will be made available by the authors, without undue reservation.

## Author Contributions

SZ and DS organized ideas and drafted the manuscript. J-DG, YY, and MX provide significant advice for the manuscript and revised the manuscript. All authors contributed to the article and approved the submitted version.

## Conflict of Interest

The authors declare that the research was conducted in the absence of any commercial or financial relationships that could be construed as a potential conflict of interest.

## Publisher’s Note

All claims expressed in this article are solely those of the authors and do not necessarily represent those of their affiliated organizations, or those of the publisher, the editors and the reviewers. Any product that may be evaluated in this article, or claim that may be made by its manufacturer, is not guaranteed or endorsed by the publisher.

## Footnotes

1
webofscience.com
